# Determination of key residues in MRGPRX2 to enhance pseudo-allergic reactions induced by fluoroquinolones

**DOI:** 10.1038/s41598-022-10549-6

**Published:** 2022-04-22

**Authors:** Eri Hamamura-Yasuno, Junya Matsushita, Seiji Sato, Takashi Shimada, Yoshimi Tsuchiya, Kazunori Fujimoto, Kazuhiko Mori

**Affiliations:** 1 Medicinal Safety Research Laboratories, Daiichi Sankyo Co., Ltd., 1-16-13 Kitakasai, Edogawa-ku, Tokyo 134-8630 Japan; 2 Modality Research Laboratories, Daiichi Sankyo Co., Ltd., 1-2-58 Hiromachi, Shinagawa-ku, Tokyo 140-8710 Japan; 3 Organic & Biomolecular Chemistry Department, Daiichi Sankyo RD Novare Co., Ltd., 1-16-13 Kitakasai, Edogawa-ku, Tokyo 134-8630 Japan; 4 Daiichi Sankyo RD Novare Co., Ltd., 1-16-13 Kitakasai, Edogawa-ku, Tokyo 134-8630 Japan

**Keywords:** Acute inflammation, Drug development, Risk factors, Inflammation

## Abstract

MAS-related G protein-coupled receptor X2 (MRGPRX2), expressed in human mast cells, is associated with drug-induced pseudo-allergic reactions. Dogs are highly sensitive to the anaphylactoid reactions induced by certain drugs including fluoroquinolones. Recently, dog MRGPRX2 was identified as a functional ortholog of human MRGPRX2, with dog MRGPRX2 being particularly sensitive to fluoroquinolones. The aim of this study was to determine key residues responsible for the enhanced activity of fluoroquinolone-induced histamine release associated with MRGPRX2. Firstly, a structure model of human and dog MRGPRX2 was built by homology modeling, and docking simulations with fluoroquinolones were conducted. This model indicated that E164 and D184, conserved between human and dog, are essential for the binding to fluoroquinolones. In contrast, F78 (dog: Y) and M109 (dog: W) are unconserved residues, to which the species difference in fluoroquinolone sensitivity is attributable. Intracellular calcium mobilisation assay with human MRGPRX2 mutants, in which residues at positions 78 and 109 were substituted to those of dog MRGPRX2, revealed that M109 and F78 of human MRGPRX2 are crucial residues for enhancing the fluoroquinolone-induced histamine release. In conclusion, these key residues have important clinical implications for revealing the mechanisms and predicting the risks of fluoroquinolone-mediated pseudo-allergic reactions in humans.

## Introduction

Drug-induced pseudo-allergic reaction is an IgE-independent adverse effect frequently observed upon the administration of drugs including antibacterial agents and peptidergic drugs, leading to histamine release, inflammation, pruritus, and airway constriction^1,2^. In severe cases, hypotension and shock-like syndrome are observed^3^.

MAS-related G protein-coupled receptor X2 (MRGPRX2), one of the class A GPCRs, has been identified as an essential receptor for pseudo-allergic reactions, and various drugs including fluoroquinolones activate Gi or Gq proteins via MRGPRX2 and induce mast cell degranulation^4^. Recently, the binding sites of ligands such as substance P, hemokinin-1, and icatibant in MRGPRX2 were reported by several research groups^5–7^. However, fluoroquinolone-binding sites of MRGPRX2 have not been comprehensively determined.

MRGPRX2 and its orthologs have been detected in not only humans but also rodents, primates, and dogs^4,8,9^. In rodents, Mrgprb2 and Mrgprb3 have been identified as the mouse and rat orthologs of human MRGPRX2, respectively^10,11^. However, the Mrgprb2 mutant mouse may not be a suitable model to screen drugs with pseudo-allergic potential for human use because the responses of Mrgprb2-expressing cells against substance P or fluoroquinolones are markedly weaker than those of human MRGPRX2-expressing cells^4^. Dogs, one of the most frequently used laboratory animals in drug development, are known to be highly sensitive to the anaphylactoid reactions induced by certain drugs including fluoroquinolones^12–15^. In fact, treatment with neuromuscular blocking agents or fluoroquinolones induces severe hypotension or shock-like syndrome accompanied by elevation of blood histamine concentrations in dogs^16–19^. On the other hand, a 30- to 100-fold higher dose of the drugs is needed to induce these symptoms in rats^14^. Therefore, dogs would be a suitable animal model to identify the risks or investigate the mechanisms of drug-induced anaphylactoid reactions. Recently, we identified that dog MRGPRX2 is a functional ortholog of human MRGPRX2, with dog MRGPRX2 being highly sensitive to fluoroquinolones^20^. However, the mechanism underlying its enhanced activity in fluoroquinolone-induced histamine release has not been clarified.

The present study was designed to identify key residues associated with fluoroquinolone-induced histamine release inducing the above-mentioned species difference in fluoroquinolone sensitivity. Firstly, a docking simulation with fluoroquinolones was conducted using a structure model of human and dog MRGPRX2 established by homology modeling. Comparison of amino acids around the predicted ligand binding pocket between human and dog indicated that two residues, F78 and M109, might play an important role in fluoroquinolone sensitivity. Then, HEK293 cells transfected with human MRGPRX2 mutants, in which amino acids at positions 78 and 109 were replaced by those in dog MRGPRX2 (F78Y, M109W, and F78Y/M109W), were treated with fluoroquinolones [ciprofloxacin (CPFX), gatifloxacin (GFLX), levofloxacin (LVFX), and pazufloxacin (PZFX)] and subjected to an intracellular calcium mobilisation assay, to assess Gq-coupled receptor activity. Determination of key residues associated with fluoroquinolone-induced histamine release would be clinically valuable for elucidating the mechanisms and predicting the risks of drug-induced pseudo-allergic reactions to fluoroquinolones in humans.

## Results

### Homology modeling of human MRGPRX2 and docking of fluoroquinolones

**Figure 1:**
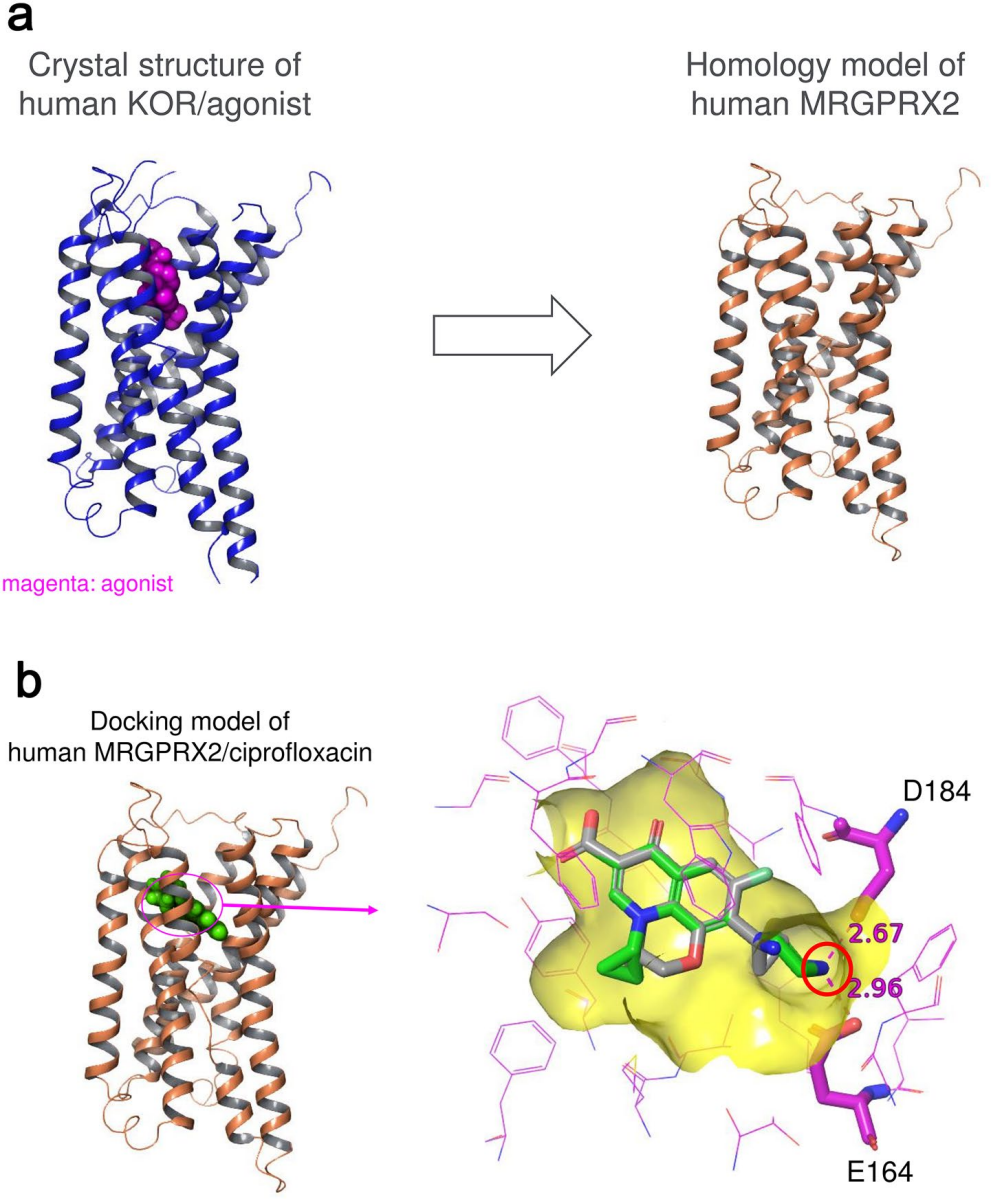
Homology modeling of human MRGPRX2 and docking simulation with fluoroquinolones. (**a**) Homology modeling of human MRGPRX2. A model of human MRGPRX2 was built with Prime version 4.5 using the crystal structure of agonist-bound human kappa opioid receptor (KOR) as a template. (**b**) Docking model of human MRGPRX2 with ciprofloxacin (CPFX, green) or pazufloxacin (PZFX, gray). A basic substituent at the 7 position of CPFX is highlighted by a red circle. The docking study incorporating the induced fit effect was performed with the Induced Fit Docking (IFD) algorithm from Schrödinger. The docking model of PZFX was constructed by superimposition with CPFX.

To perform docking simulations with fluoroquinolones, a homology model of human MRGPRX2 was built because no crystal structure information was available. As one of the closest homologs in the Protein Data Bank (https://www.rcsb.org), human KOR (PDB accession id: 6B73, agonist-bound form) was used as a template for the modeling (Fig. 1a). Next, a docking simulation incorporating the induced fit effect with fluoroquinolones was performed. In this simulation, fluoroquinolones binding to human MRGPRX2 were simulated at the same site of the agonist in PDB 6B73. The top-ranked pose is shown in Fig. 1b. In the docking model with CPFX, a fluoroquinolone that induces histamine release by mast cells^14,21^, characteristic salt bridges were found between a basic substituent at the 7 position of CPFX and acidic side chains of E164 and D184 in human MRGPRX2. On the other hand, in the model with PZFX, a fluoroquinolone that does not induce histamine release by mast cells^22^, a side chain at the 7 position was located at a more distant location from E164 and D184 than in CPFX because of the difference in position of the terminal nitrogen between these two fluoroquinolones. Thus, E164 and D184 in human MRGPRX2 were considered as essential residues involved in molecular interactions associated with the activation of MRGPRX2 by fluoroquinolones.

### Amino acid sequence alignment and comparison of human and dog MRGPRX2

**Figure 2:**
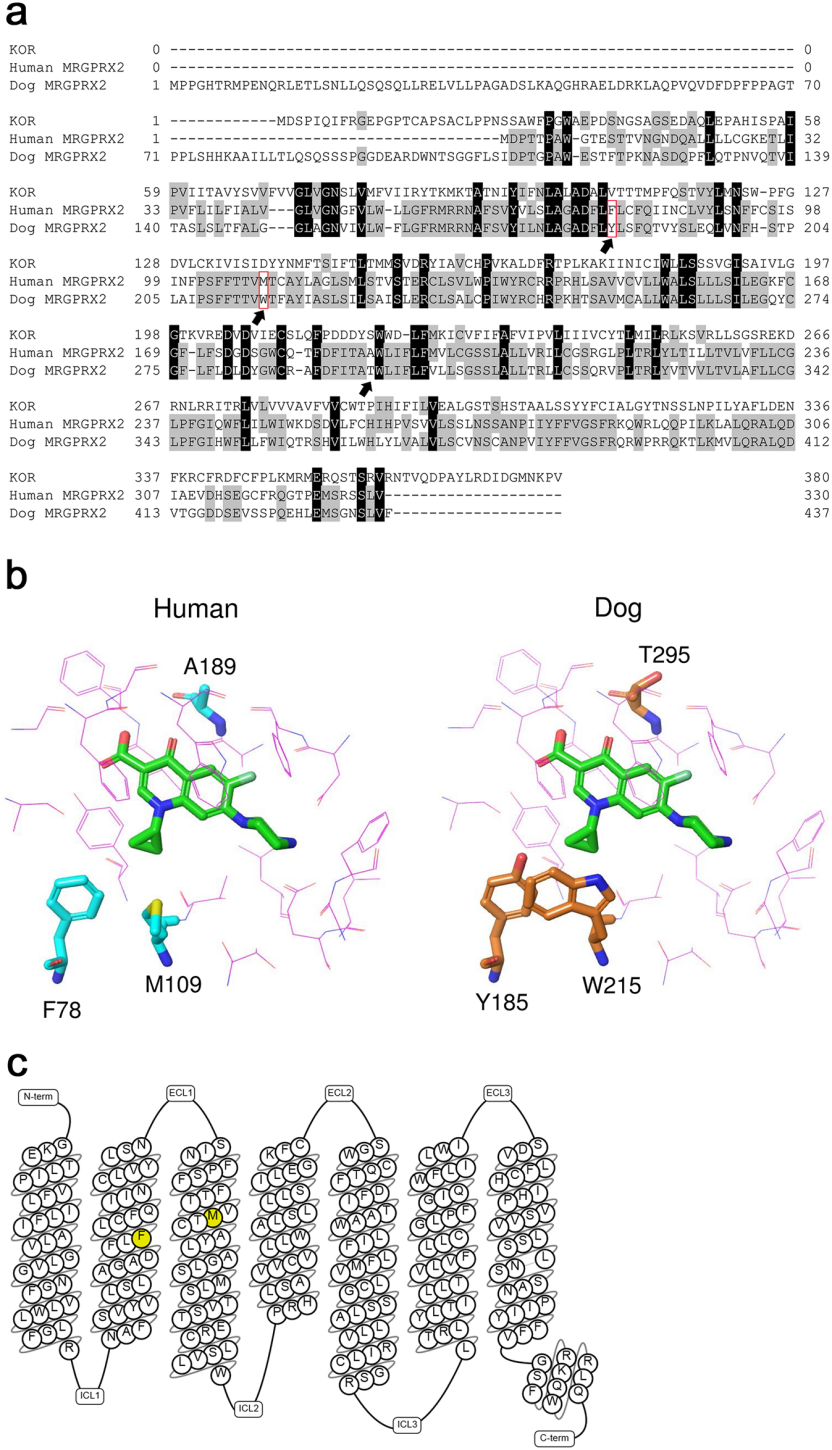
Comparison of human and dog MRGPRX2. (**a**) Alignment of human kappa opioid receptor (KOR), human MRGPRX2, and dog MRGPRX2. Amino acid sequence alignment was carried out using GENETYX-SV/RC Ver.13.1.1. Arrows indicate unconserved residues between human and dog MRGPRX2 around the estimated ligand binding pocket. (**b**) Homology modeling of human and dog MRGPRX2 bound with ciprofloxacin (CPFX, green). Unconserved residues between human and dog MRGPRX2 within 5 Å around CPFX are highlighted in cyan (human) and orange (dog). (**c**) Snake diagram of human MRGPRX2. The residues at which dog-type mutations were introduced are marked in yellow. Generated using tools of gpcrdb.org.

The result of aligning human and dog MRGPRX2 is shown in Fig. 2a. Typical motifs of class A GPCRs such as DRY motif and cysteine in transmembrane (TM)-3 were conserved in KOR, while they were not conserved in both human and dog MRGPRX2. E164 (dog: 270) and D184 (dog: 290), which were thought to be involved in key molecular interactions with fluoroquinolones as mentioned above, were shared between human and dog MRGPRX2. Next, a dog MRGPRX2 structure model was built by swapping amino acids of human MRGPRX2 constructed as described above. To identify key residues associated with a species difference, residues around the predicted binding pocket of fluoroquinolones were compared between human and dog MRGPRX2. Among the 21 residues within 5 Å around CPFX docked in human and dog MRGPRX2, only three amino acids (F78, M109, and A189 in human MRGPRX2) were unconserved residues between human and dog (Fig. 2b); the remaining residues (86%) were conserved. Among these three residues, F78 (dog: Y185) and M109 (dog: W215) in human MRGPRX2 were predicted to play some roles in the interaction with its ligands because the side chains of these two residues are oriented towards CPFX, and likely to affect it. Therefore, we selected the residues F78 and M109, located in TM2 and TM3, and constructed human MRGPRX2 mutants in which “dog-type” mutations were introduced (Fig. 2c).

### Functional assay of human MRGPRX2 mutants

**Figure 3:**
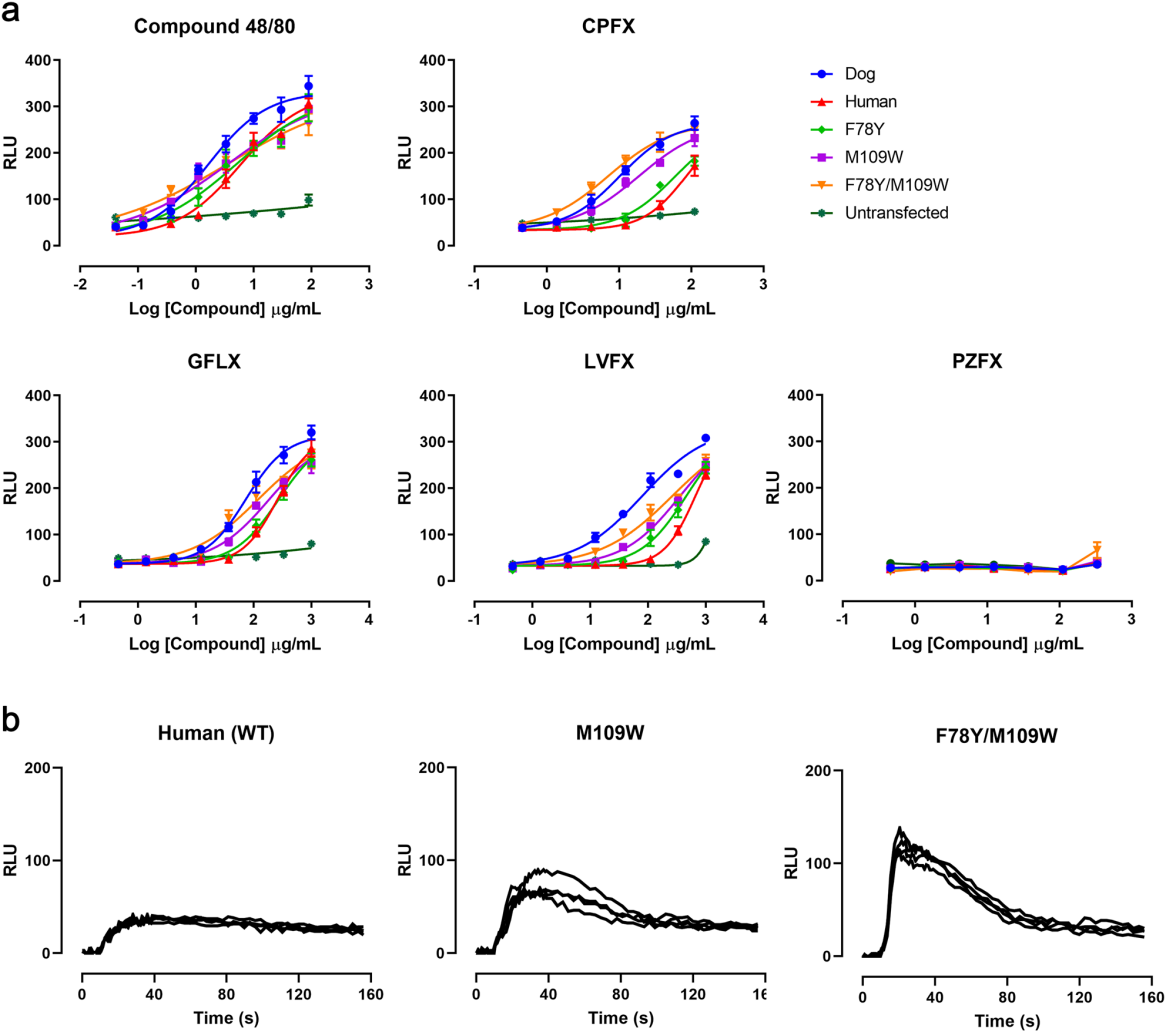
Effects of test articles on changes in intracellular calcium levels in cells expressing human MRGPRX2 or its mutant. HEK293 cells transiently transfected with dog MRGPRX2, human MRGPX2, or a mutant of human MRGPRX2 exposed to compound 48/80 or fluoroquinolones [ciprofloxacin (CPFX), gatifloxacin (GFLX), levofloxacin (LVFX), and pazufloxacin (PZFX)]. (**a**) Dose-dependent responses of intracellular calcium levels. Data are presented as the mean ± S.D. of quadruplicate assays. The four-parameter sigmoidal model was used for curve-fitting. Untransfected HEK293 cells were used as a negative control. (**b**) Time-course changes of intracellular calcium levels in cells expressing human MRGPRX2 WT or its mutant. Traces show representative intracellular calcium fluctuation following exposure to CPFX (4.1 µg/mL). Test articles were perfused from 10 s. *RLU* relative light units, *s* second.

To determine whether the dog-type human MRGPRX2 mutants were associated with increased responses, intracellular calcium mobilisation against fluoroquinolones was evaluated. For this evaluation, three types of expression vector, F78Y and M109W (single mutations), and F78Y/M109W (double mutation), were constructed. HEK293 cells were transiently transfected with these human MRGPRX2 mutants and evaluated for reactivity to compound 48/80, a typical histamine releasing agent, and fluoroquinolones (CPFX, GFLX, LVFX, and PZFX). In the mutant F78Y, slightly increased reactivity was observed against treatment with CPFX and LVFX compared with the case for wild type (WT) human MRGPRX2 (Fig. 3a). On the other hand, in the mutants M109W and F78Y/M109W, significantly increased responses to compound 48/80, CPFX, GFLX, and LVFX were observed from lower concentrations than for WT (Fig. 3a,b). In particular, the reactivity of the double mutant F78Y/M109W against CPFX was equal to or greater than that of dog MRGPRX2. The EC_50_ of CPFX in F78Y/M109W was *ca*. one-eighth that of the WT (Table 1). The order of the EC_50_ values was compound 48/80 < CPFX < LVFX < GFLX, which was the same for all of the mutants. PZFX, which does not induce histamine release by mast cells^22^, did not induce intracellular calcium mobilisation in any of the cells.

**Table 1:** Half-maximum effective concentration (EC_50_) values of test articles on changes in intracellular calcium levels in cells expressing MRGPRX2 or its mutant.

Test article	EC_50_ (µg/mL) ± SD (*P* value)				
	Human				Dog
	Wild type	F78Y	M109W	F78Y/M109W	Wild type
Compound 48/80	5.4 ± 0.57	4.8 ± 2.1 (*P* = 0.60)	2.6 ± 1.0** (*P* = 0.0028)	1.3 ± 0.69** (*P* < 0.001)	2.1 ± 0.93** (*P* < 0.001)
CPFX	47 ± 6.1	33 ± 4.7* (*P* = 0.010)	20 ± 8.4** (*P* = 0.0018)	6.1 ± 1.8** (*P* < 0.001)	12 ± 1.9** (*P* < 0.001)
GFLX	360 ± 160	250 ± 69 (*P* = 0.24)	100 ± 16* (*P* = 0.018)	74 ± 27* (*P* = 0.013)	97 ± 37* (*P* = 0.019)
LVFX	650 ± 330	500 ± 350 (*P* = 0.57)	210 ± 36* (*P* = 0.038)	180 ± 140* (*P* = 0.038)	90 ± 47* (*P* = 0.015)

Statistical significance was determined by unpaired t-test. Data are represented as mean ± S.D. of quadruplicate assays.

*CPFX* ciprofloxacin, *GFLX* gatifloxacin, *LVFX* levofloxacin.

*P < 0.05, **P < 0.01 vs. wild type human MRGPRX2.

## Discussion

In the present study, we identified M109 and F78 in human MRGPRX2 as key residues associated with fluoroquinolone-induced histamine release inducing the difference in fluoroquinolone sensitivity between human and dog. Introducing dog-type mutations into human MRGPRX2 markedly enhanced responses to fluoroquinolones. Our results provide important insights into the mechanism behind fluoroquinolone-induced histamine release in dogs, which are highly sensitive to these drugs. Moreover, the results suggest the mechanisms behind MRGPRX2-related hypersensitivity in humans.

Reddy et al. and Lansu et al. identified E164 and D184 of human MRGPRX2 as essential residues for the activation of MRGPRX2 by substance P and opioids^5,6^. These two amino acids are negatively charged residues that were found to interact with cationic opioid ligands^6^. Additionally, it was demonstrated that replacing E164 with a positively charged residue resulted in the loss of responses to substance P^6^. In contrast, the response to LL-37 or dynorphin was not lost even in the same mutant, suggesting that different ligands might interact with different amino acids around the predicted ligand-binding pocket^6^. In this study, our docking model suggested that E164 and D184 would be essential for MRGPRX2 activation associated with fluoroquinolones because of the characteristic interactions between CPFX and E164/D184. We previously reported that a basic substituent at the 7 position of the fluoroquinolone ring may be associated with histamine release^14^. Consistent with that report, a terminal basic nitrogen of piperazine at the 7 position of the fluoroquinolone ring, which is present in the three fluoroquinolones used in this study (CPFX, GFLX, and LVFX), was predicted to form interactions such as a salt bridge or a hydrogen bond with acidic side chains of E164 and D184. On the other hand, in PZFX, which does not induce histamine release by mast cells^22^, no salt bridge or hydrogen bond was formed because of the greater distance between the nitrogen and E164/D184. The difference between CPFX and PZFX in our MRGPRX2 docking model was consistent with their distinct histamine release potential in vivo/vitro^17,22^. However, these residues were not considered to be a key factor contributing to the enhanced activity to fluoroquinolones because they were conserved between human and dog.

Human MRGPRX2 is considered as “an atypical opioid receptor”^5^; many of the motifs conserved in class A GPCRs are not found in human and dog MRGPRX2. While the homology of amino acid sequences between human and dog MRGPRX2 was 62%^20^, residues around the predicted ligand-binding site were found to be well conserved; only three amino acids differed. In the functional assay with “dog-type” human MRGPRX2 mutants, M109W and F78W/M109W showed markedly increased responses compared with the WT. In particular, the double mutant F78Y/M109W appeared to demonstrate a greater response than dog MRGPRX2 against CPFX. These results clearly suggest that M109 (dog: W215) and F78 (dog: Y185) of human MRGPRX2 are key residues contributing to the enhanced responses to fluoroquinolones.

Introducing a mutation into a GPCR often affects its basal activity or ligand sensitivity^23,24^. In this study, human MRGPRX2 mutants did not exhibit changes of basal activity compared with WT in the calcium mobilisation assay, suggesting that these mutations did not affect the constitutive activity of MRGPRX2. In contrast, the mutants M109W and F78Y/M109W demonstrated a left shift of the dose–response curves and decreased EC_50_ values compared with WT, indicating that the mutations evoked increases in interactions with its ligands or binding affinity. According to the Ballesteros-Weinstein GPCR numbering method, M109 and F78 in human MRGPRX2 are indicated as 3 × 32 and 2 × 53, respectively^25,26^. Amino acids located at 3 × 32 in TM3 have been reported to be involved in interactions with ligands in some GPCRs^27,28^, and are listed as being among the most important residues that frequently interact with ligands^29^. In the human MRGPRX2 mutant M109W, the change to this bulky aromatic residue at position 3 × 32 might affect the size of the ligand binding pocket and result in different responses. In addition, marked enhancement was induced by co-mutation with F78Y compared with single mutant M109W. Phenylalanine is closely related to tyrosine, which just has an additional hydroxyl group. Therefore, as for the double mutant F78Y/M109W, the hydroxy group in tyrosine would generate a different interaction favorable to the fluoroquinolone binding and resulted in enhanced responses.

Among the fluoroquinolones used in this study, CPFX markedly induced an enhanced response to F78Y/M109W compared with GFLX and LVFX. Although the chemical structures of CPFX and GFLX are almost the same, CPFX does not have a methoxy group at the 8 position of the fluoroquinolone ring. In addition, M109 is located adjacent to the 1 and 8 positions of the fluoroquinolone ring in the human MRGPRX2 docking model. Thus, the difference of chemical structure at the 8 position of the fluoroquinolone ring would have resulted in the different levels of enhancement in responses of F78Y/M109W. As for the order of EC_50_, human MRGPRX2 WT and all of the mutants generated in this study showed the same order of fluoroquinolones: CPFX < GFLX < LVFX. In contrast, the EC_50_ of LVFX was similar to or lower than that of GFLX in dog MRGPRX2 WT, suggesting that the ligand selectivity to these fluoroquinolones differs between human and dog MRGPRX2. Because the order did not change even in the mutants constructed in this study, the difference in ligand selectivity may arise from regions other than F78 and M109. Because the residues around the ligand binding pocket of MRGPRX2 are highly conserved between human and dog, comparison between human and dog MRGPRX2 including the site responsible for G-protein coupling, such as intracellular loops, may be needed to clarify the mechanism behind the different ligand selectivity between human and dog.

Non-synonymous single-nucleotide polymorphisms (SNPs) would affect the responses to ligands, associating with hypersensitivity against drugs in some patients^30,31^. Although a number of naturally occurring missense variants of human *MRGPRX2* have been reported, most of the variants showed similar or decreased responses compared with WT^32^. On the other hand, Chompunud Na Ayudhya et al. have reported that human MRGPRX2 mutants at the carbonyl terminus, which are associated with receptor phosphorylation and desensitisation, showed enhanced responses to substance P^33^. In the present study, we constructed human MRGPRX2 mutants at the TM domain, which is associated with ligand binding, and these mutants demonstrated enhanced responses to fluoroquinolones compared with WT. Our results indicate that missense mutations in F78 or M109 of human MRGPRX2 would induce enhanced responses to certain fluoroquinolones including CPFX in humans. With regard to human MRGPRX2 variants, F78L and V108A are found in gnomAD as naturally occurring missense mutations located at or near positions F78 and M109, respectively. The mutant F78L was reported not to alter the activity against hemokinin-1, substance P, icatibant, and human β-defensin-3^7^, generally consistent with the results of this study. However, it should be considered that the mutation at F78 may induce a greatly enhanced response when it is accompanied by mutation at M109. No reports have been published with regard to V108A’s function. Further analysis of SNPs located around F78 or M109 would provide important information to investigate MRGPRX2-related hypersensitivity.

In summary, we focused on dog MRGPRX2, which is sensitive to fluoroquinolones compared with human MRGPRX2, and identified key residues associated with fluoroquinolone-induced histamine release that explain this species difference. Our results have important clinical implications for revealing the mechanism behind fluoroquinolone-mediated pseudo-allergic reactions and assessing the risk of them in humans.

## Materials and methods

### Homology modeling and docking simulation

A homology model of human MRGPRX2 (UniProt accession id: Q96LB1) was built with Prime version 4.5 (Schrödinger, LLC, New York, NY^34,35^; using a crystal structure of agonist-bound human kappa opioid receptor (KOR, PDB accession id: 6B73) as a template. For the modeling, we used the same method of amino acid sequence alignment as in a previous study^5^. A docking study with fluoroquinolones incorporating the induced fit effect was performed with the Induced Fit Docking (IFD) algorithm from Schrödinger^36–38^.

### Amino acid sequence alignment

Amino acid sequence alignment of human MRGPRX2 (accession No. NP_001290544), dog MRGPRX2 (accession No. XP_005633869), and KOR (accession No. AAC50158) was carried out using GENETYX-SV/RC Ver.13.1.1 (Genetyx Corporation, Tokyo, Japan).

### Reagents

Compound 48/80 was purchased from Sigma-Aldrich Co. LLC (St. Louis, MO). CPFX and LVFX were obtained from Fujifilm Wako Pure Chemical Corporation (Osaka, Japan), and GFLX and PZFX were obtained from LKT Laboratories Inc. (St. Paul, MN).

### Site-directed mutagenesis of human MRGPRX2

The WT human MRGPRX2 gene in pcDNA3.1(+) was used as a template. Point mutations of F78Y, M109W, and F78Y/M109W were introduced with PrimeSTAR Max DNA Polymerase (Takara Bio Inc., Kusatsu, Japan) and primers (see Table 2). DH5α competent cells generated by Mix & Go *E. coli* Transformation kit (Zymo Research Corp., Irvine, CA) were transformed with the plasmids and cultured. The plasmids were extracted and sequenced by Daiichi Sankyo RD Novare Co., Ltd. (Tokyo, Japan).

**Table 2:** Primers used in construction of human MRGPRX2 mutants.

F78Y inversion	FP: 5′-TTCCTGTACCTTTGCTTCCAGATAATT-3′ RP: 5′-GCAAAGGTACAGGAAATCGGCGCCTGC-3′
M109W inversion	FP: 5′-ACTGTTTGGACCTGTGCATATCTGGCC-3′ RP: 5′-ACAGGTCCAAACAGTAGTGAAAAAACT-3′

### Transfection of HEK293 cells with MRGPRX2 and its mutants

HEK293 cells obtained from the JCRB Cell Bank (Osaka, Japan) were transiently transfected with MRGPRX2 and its mutants. Lipofectamine 2000 Reagent (Thermo Fisher Scientific Inc., Waltham, MA) and pcDNA3.1(+) containing each gene were diluted and mixed using Opti-MEM I Reduced Serum Medium (Thermo Fisher Scientific Inc.) to prepare lipid-DNA complexes (final concentrations: lipofectamine 2.5 µL/mL and DNA 2500 ng/mL). HEK293 cells were detached using TrypLE Express (Thermo Fisher Scientific Inc.) and prepared to 7 × 10^5^ cells/mL with the lipid-DNA complex. Thereafter, 25 µL of cells (1.75 × 10^4^ cells/well) were seeded per well in 384-well flat-bottomed plates (Corning Incorporated, Corning, NY) and incubated overnight at 37 °C under 5% CO_2_ conditions. Cells treated with plasmid-free lipid solution were used as a negative control (untransfected cells).

### Calcium mobilisation assay

Test articles were dissolved in Hanks’ balanced salt solution (HBSS, pH 7.4; Thermo Fisher Scientific Inc.) supplemented with 20 mM hydroxyethylpiperazine-N′-2 ethanesulfonic acid (HEPES; Sigma-Aldrich Co. LLC) and 0.05 vol% bovine serum albumin (BSA; Sigma-Aldrich Co. LLC). The highest concentration of fluoroquinolones was set at 1000 µg/mL based on previous reports, at which the test substances induced marked intracellular calcium mobilisation in MRGPRX2-expressing HEK293 cells^10,20^ or caused histamine release in rat or human mast cells^14,17,21^. Intracellular calcium levels were analysed using Calcium Kit II-iCellux (Dojindo Molecular Technologies, Inc., Kumamoto, Japan), in accordance with the manufacturer’s instructions. HEK293 cells (1.75 × 10^4^ cells/well) were loaded with 1.25 mM probenecid and calcium probe for 45 min at 25 °C. Changes in fluorescence intensities between before and after addition of the test articles were measured over time using FLIPR Tetra (Molecular Devices, LLC, Sunnyvale, CA) with excitation at 470–495 nm and emission at 515–575 nm. The test articles were added 10 s after beginning the measurements. The data were analysed using ScreenWorks (Molecular Devices, LLC, Version 3.2.0.14) to determine the difference between maximal and minimal fluorescence intensity (max–min). As CPFX at 333 and 1000 µg/mL induced nonspecific increases in intracellular calcium levels in untransfected cells, these data were excluded from the analysis. All experiments were performed in quadruplicate.

### Statistical analysis

Data are presented as the mean ± S.D. for calcium mobilisation. Half-maximal effective concentration (EC_50_) of each test article used in the calcium mobilisation assay was calculated from individual Emax and E0 for each variant using the four-parameter sigmoidal model. Statistical significance (*P* < 0.05) was determined by unpaired t-test. These analyses were performed using GraphPad Prism 7.03 (GraphPad Software, La Jolla, CA).

## Data Availability

All data generated or analysed during this study are included in this published article.
